# Ingleby Lectures: Lecture II.—The Injuries of Bones

**Published:** 1891-04-18

**Authors:** 


### SURGERY.

### INGLEBY LECTURES.—Lecture II.

#### The Injuries of Bones.

Mr. Lloyd prefaced his remarks on "The Injuries and Diseases of Bones" by saying he was going to call attention chiefly to those conditions which are frequently overlooked, viz.:—

(1) Fracture of the Clavicle.—It is a frequent occurrence for children to be brought to hospital surgeons with swellings at the root of the neck, which at once can be recognised as caused by the callous thickening round an unsuspected fractured clavicle. The mother will often tell you the child has had a fall and was taken to a doctor who had said there was very little the matter. He has overlooked the fracture—and why? Because he has not followed a rule it is invariably wise to follow, viz., when a child is brought to you who has fallen and hurt its arm, have it stripped and examine the whole upper limb from the sternum to the finger-tips. Your attention is frequently drawn to some other part of the arm, as the elbow, where there is nothing the matter. A tender spot in the middle of a clavicle of a baby probably means fracture. *Treatment.*—The old plan is the best, viz., the axillary pad and the bandage, firmly binding the arm to the side, with the forearm and hand bent across the chest and the elbow supported by a few turns carried over the opposite shoulder.

(2) Fractures of the Humerus.—The fractures most commonly overlooked in this bone are those of its lower extremity. A surgeon often has cases brought to him several weeks after injury, because of the patient's inability to fully extend and flex the elbow-joint. Many of these cases have been under professional treatment and regarded as sprains. Why are these fractures overlooked? Because they are not thoroughly examined immediately after injury, owing to the cries of the child and the sympathetic entreaties of the mother for you to leave it alone—at any rate, till to-morrow. If you yield, effusion results, and prevents you making an accurate diagnosis. Unless you can absolutely satisfy yourself as to the exact nature of the injury, give an anæsthetic in all these cases. A week or two after injury the diagnosis is made easy by (1) a thickening about the lower end of the humerus, mostly in front of the internal condyle; (2) limited movement of the joint. Another good diagnostic sign of this condition is seen in the alteration of the longitudinal axes of the humerus and ulna to each other; in the normal condition the ulna is hinged into the humerus at a slight angle outwards; in any disturbance in the planes the lower articular end of the humerus carries the lower end of the ulna to one or the other side of its normal position.

As Regards Treatment.—Mr. Lloyd is in the habit of using a millboard-box splint, which is worn for four or five weeks, and then when all reparative action has passed away, passive movement, if necessary with an anæsthetic, is employed.

(3) Fractures of the Radius.—Fractures of the lower end of the radius are often treated as sprains. Especially is this so in a class of cases where the amount of break falls short of completely detaching the lower end of the bone. In these cases crepitus, deformity, adventitious mobility may all be absent, but if the nature of the accident and the subjective sensations of the patient point to a serious injury about the wrist-joint, it is wise to treat the case as you would an undoubted fracture. Another interesting condition is that where after an injury to the lower end of the radius there occurs retarded development of the bone, patients come complaining of a "growing out" of a bone at the inner side of the lower end of the forearm; this is caused by the hand being pushed over by the ulna in consequence of retarded growth on the part of the radius.

(4) Fractures of the Femur.—Mr. Lloyd described two cases of a rare kind of fracture of the lower end of the femur which had been overlooked. In both cases his attention was especially called to a sharp bony prominence which had first been seen three weeks after the injury, on the outer side of the limb, exactly opposite the patella. After the accident the patient was unable to stand; there was pain, rapid swelling, absence of crepitus and deformity and usual mobility; the progress was very slow, and then this bony joint appeared. Examination showed shortening of one and a-half to two inches.

Diagnosis.—Oblique fracture of lower end of shaft of femur from above downwards and outwards, the lower end of the upper fragment burying itself in the vastus externus muscle, its tendon and the capsule of the joint, the upper end of the lower fragment being hidden at the back of the shaft of the bone.

For fractures of femur in children, Mr. Lloyd strongly recommends treatment by vertical extension. Its advantages are: (1) The ease with which you can change napkins, and so keep the dressings clean. (2) The perfect comfort of the position. In concluding the subject of fractures, Mr. Lloyd said he had met with cases of overlooked fracture in every bone of the body which was liable to break.

